# Extensive multifocal branch duct IPMN of the pancreas after liver transplantation: is surgery justified?

**DOI:** 10.1186/s40001-015-0117-5

**Published:** 2015-03-19

**Authors:** Vittorio Branchi, Philipp Lingohr, Winfried A Willinek, Alexander Semaan, Hui Zhou, Glen Kristiansen, Günter Klöppel, Jörg C Kalff, Nico Schäfer, Hanno Matthaei

**Affiliations:** Department of Surgery, University Hospital of Bonn, Sigmund-Freud-Str. 25, 53127 Bonn, Germany; Institute of Pathology, University Hospital of Bonn, Sigmund-Freud-Str. 25, 53127 Bonn, Germany; Department of Radiology, University Hospital of Bonn, Sigmund-Freud-Str. 25, 53127 Bonn, Germany; Center for Integrated Oncology (CIO) Cologne-Bonn, Sigmund-Freud-Str. 25, 53127 Bonn, Germany; Department of Pathology, Center for Pancreatic and Endocrine Tumors, Technical University Munich, Trogerstr. 18, 81675 Munich, Germany

**Keywords:** IPMN, Liver transplantation, Immunosuppression, Pancreatic cystic tumors

## Abstract

**Background:**

Cystic lesions of the pancreas resembling intraductal papillary mucinous neoplasms (IPMN) have been reported to develop in an increased rate following liver transplantation and immunosuppression. The cause for this possible association is thus far elusive.

**Presentation of the case:**

We report on a 60-year-old male patient who developed an extensive multicystic change of the entire pancreas, suspicious for IPMN, under follow-up after liver transplantation for secondary sclerosing cholangitis. A total pancreaduodenectomy with splenectomy was performed. The postoperative histopathological assessment revealed a multifocal branch duct IPMN of the gastric subtype showing low-grade dysplasia.

**Discussion:**

In the absence of evidence-based guidelines for the management of suspected IPMNs in liver transplant recipients, each patient’s management should be discussed in detail.

**Conclusion:**

Prospective studies may help to understand the disease and identify risk factors for malignant transformation in IPMNs after liver transplantation for treatment optimization.

## Background

Pancreatic cysts are increasingly diagnosed these days. Recent reports suggested a higher prevalence of IPMN-like cysts in patients after liver transplantation and subsequent immunosuppressive therapy. Since evidence-based guidelines are not established yet an ideal treatment is still controversially discussed sometimes causing a therapeutic dilemma.

## Case presentation

A 60-year-old man was referred to our outpatient clinic because of persisting lower back pain and hyporexia. The patient was under follow-up after liver transplantation. Seven years prior to admission, he was treated at the intensive care unit for third degree burns covering approximately 30% of his body surface. During a complicated clinical course, he developed a severe secondary sclerosing cholangitis with biliary cirrhosis and was therefore listed for a liver transplantation. In November 2006, 8 months after the accident, he underwent whole living donor liver transplantation. Subsequently, the patient received a long-term immunosuppressive therapy with Tacrolimus, maintained at a blood concentration between 4.1 and 14.59 ng/ml and Mycophenolate 500 mg every 8 h. He regularly attended follow-up at our clinic and never suffered from any complications related to the immunosuppressive treatment regimen. Routine laboratory tests including blood count, liver function tests, bilirubin, and C-reactive protein were normal. He denied alcohol consumption and drug abuse.

**Figure 1:**
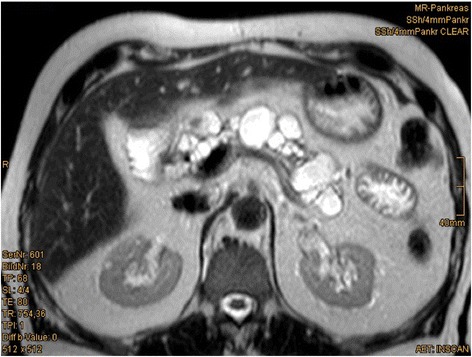
**Magnetic resonance tomography (MRT) showing a multifocal cystic lesion of the pancreas involving the whole organ.**

**Figure 2:**
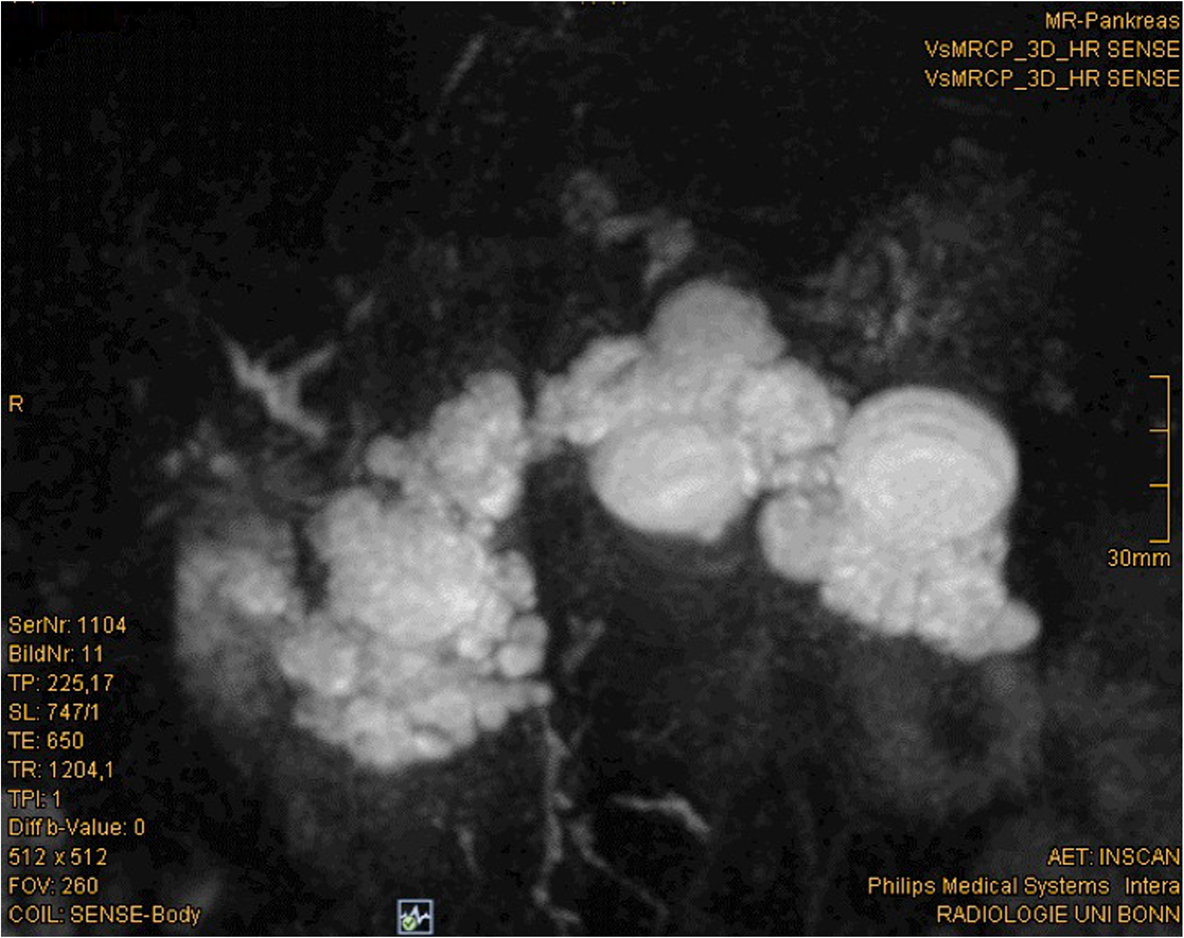
**Magnetic resonance cholangiopancreatography (MRCP) with 3D reconstruction demonstrates the multicystic, grape-like appearance of the lesion.**

**Figure 3:**
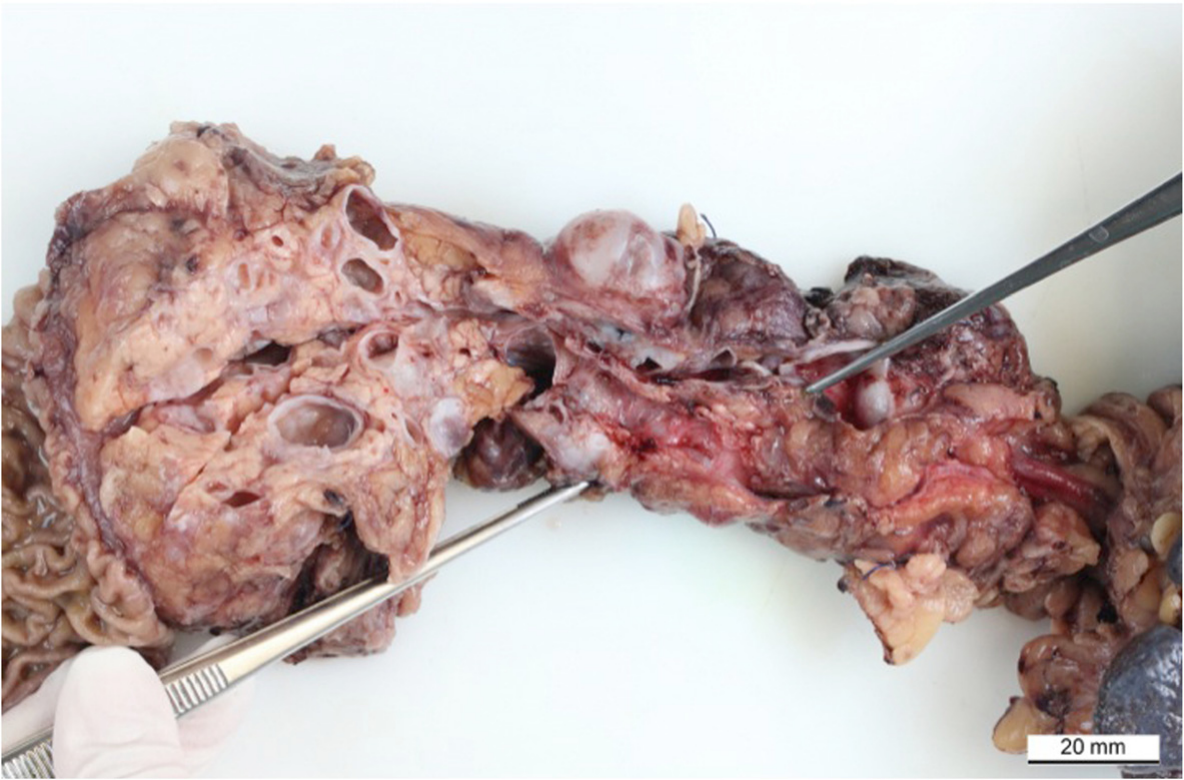
**Formalin-fixed pancreas specimen that showed multiple cystic branch duct IPMNs while the main pancreatic duct appears only minimally dilated.**

**Figure 4:**
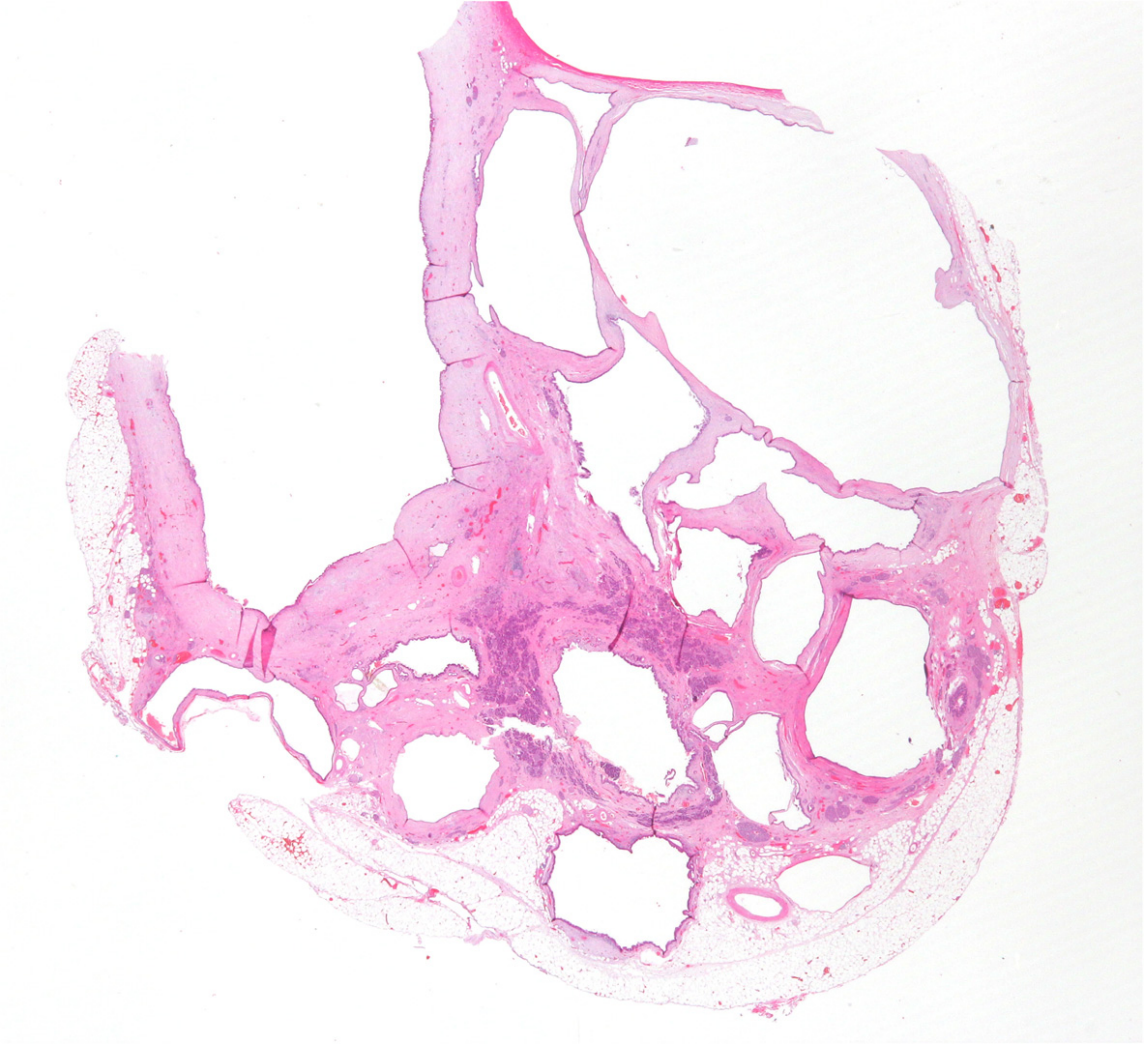
**Hematoxylin and eosin staining showed IPMN-epithelium with merely low-grade dysplasia of the gastric epithelial subtype.**

**Figure 5:**
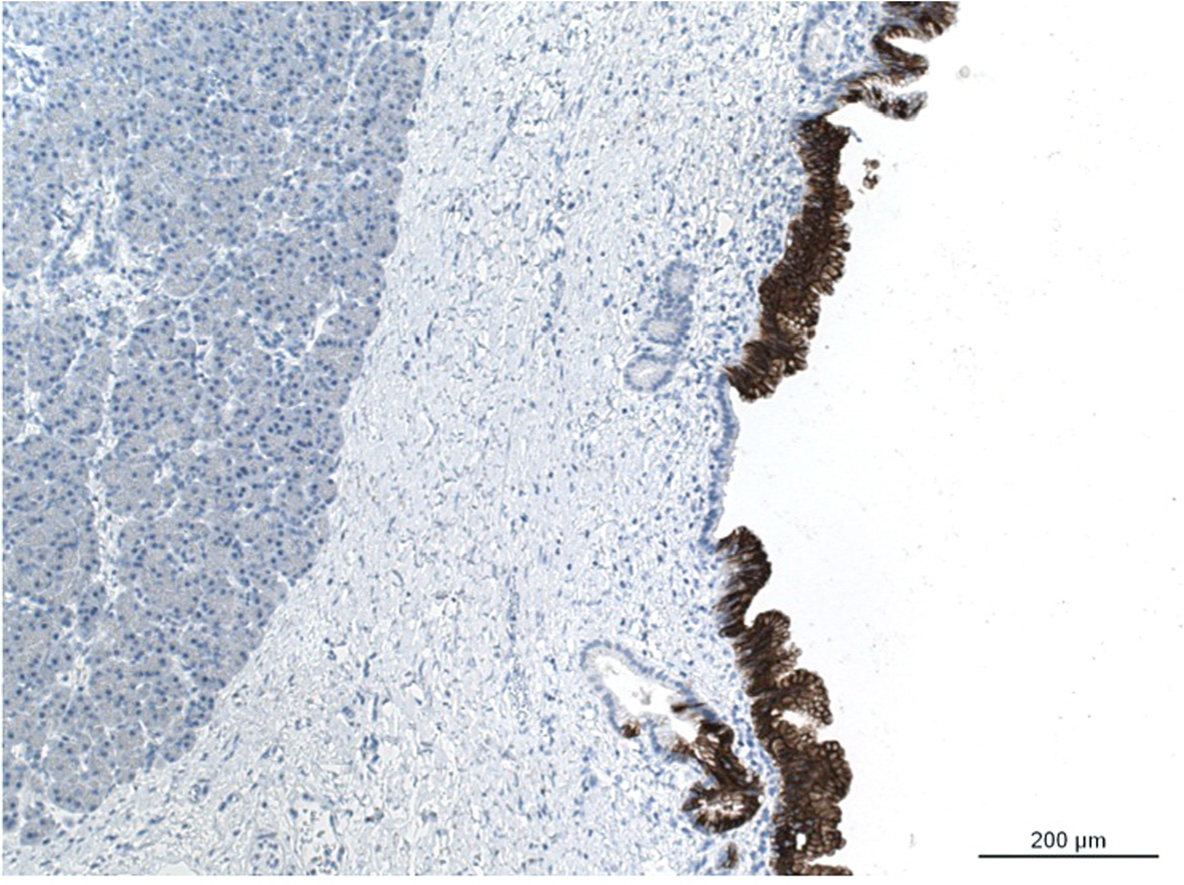
**MUC5AC-immunoihstochemical staining of the epithelium confirmed the presence of gastric-type IPMN.**

Seven years after transplantation, a sonography performed for abdominal discomfort newly diagnosed multiple cystic lesions in the pancreatic head, body, and tail, the biggest lesion measuring 3.5 cm in maximum diameter. A magnetic resonance cholangiopancreatography (MRCP) confirmed the presence of multiple pancreatic cysts involving the entire gland (Figures 1 and 2). The lesions were morphologically compatible with a multifocal branch duct type intraductal papillary mucinous neoplasms (BD-IPMN). In order to obtain a biopsy for histopathological examination, an endoscopic retrograde cholangiopancreatography (ERCP) was carried out, which revealed a communication between the cysts and the dilated main pancreatic duct. Brush cytology showed papillary epithelium suspicious for IPMN. After thorough discussion in our interdisciplinary, weekly tumor board surgical resection was recommended. In November 2013, the patient underwent a total pancreatoduodenectomy and splenectomy. The histopathological assessment revealed a multifocal BD-IPMN of the gastric subtype with low-grade intraepithelial dysplasia (Figures 3, 4, and 5). No high-grade dysplasia or associated invasive cancer was detected on a multitude of sections from the entire specimen, which were independently assessed by three experienced pathologists (HZ, GK, GK). The postoperative course was uneventful, and the patient was discharged on the 14th postoperative day. Seven months after surgery, he presented in a favorable condition without any relevant complaints.

## Discussion

This case reports the development of a multifocal branch duct type IPMN 7 years after liver transplantation and thus addresses the question regarding a causal relationship between liver transplantation and immunosuppression and the occurrence of cystic neoplasms in the pancreas. During the past few decades, the development of new immunosuppressive drugs and progress in surgical and perioperative treatment has led to a better prognosis after liver transplantation with more than half of those patients surviving for >20 years [1-3]. Development of neoplasms is a severe complication of long time immunosuppressive therapy [4]. The strong association between immunosuppression and cancer is nowadays well understood [5]. Several factors may contribute to carcinogenesis in liver transplant recipients including duration, type, and intensity of immunosuppression. As a consequence, cancer rates after liver transplantation are two- to threefold higher than in the age- and sex-matched population [6]. The most frequently observed neoplasm is post-transplant lymphoproliferative disorder (PTLD), usually observed relatively shortly after transplantation. Gastrointestinal cancers, in contrast, seem to be related to long-term immunosuppression [7]. In a recent large population-based study, the standardized incidence ratio (SIR) for *de novo* pancreatic cancer after liver transplantation was between 2.31 and 3.6 [8-11]. The literature about the onset of pancreatic cystic neoplasms after liver transplantation is, however, sparse. Girometti *et al*. showed that the prevalence of pancreatic cysts in patients after liver transplantation according to MRCP imaging was 59.6% [12]. Interestingly, 42.9% of the detected lesions showed features reminiscent of IPMN. These rates are considerably higher than in the general population although the prevalence of pancreatic cystic lesions in the literature varies considerably depending on the diagnostic method used. Reported numbers range from 0.2% on abdominal ultrasound [13], 2.6% on computed tomography (CT) [14] to most representative 25% if considering postmortem necropsies in individuals without known pancreatic disease [15]. Thus far, it can only be speculated if the apparently higher incidence of pancreatic cystic lesions following liver transplantation is influenced by pre- or post-transplantation factors, such as preoperative pancreatic damage (for example, alcohol consumption or chronic pancreatitis) or postoperative immunosuppressive therapy. The clinical situation is certainly rare, and accordingly, the underlying pathomechanisms are poorly understood and no evidence-based treatment has been established. It is also unknown if IPMNs developing in patients after liver transplantation and immunosuppression have the same biologic behavior as ‘sporadic’ IPMNs.

There is an agreement that indication for abdominal surgery should be carefully evaluated in patients with liver transplants. Apart from possible severe adhesions due to prior major abdominal surgery, these patients have a high risk for infectious diseases with subsequent septic organ dysfunction. Nevertheless, in case of an IPMN with its potential to develop into a lethal invasive carcinoma, resection may be the only chance to prevent the patient from this deadly disease. The decision to perform a total pancreatectomy in our patient was based on the following considerations: our patient had a multifocal cystic lesion involving the branch ducts of the entire pancreatic gland. Preoperative imaging also suspected a partial involvement of the main pancreatic duct, which appeared dilated up to 10 mm, a ‘worrisome feature’ implying resection according to the Sendai consensus criteria. Additionally, our patient suffered from abdominal discomfort as potentially IPMN-associated symptom. With respect to the usually reduced life expectancy subsequent to liver transplantation, it is, however, questionable whether the Sendai criteria should be equally strictly applied in this patient population. According to the American Pancreatic Association (APA) international consensus guidelines in their latest version, surgical resection should be recommended in patients with symptomatic cysts and in case of main pancreatic duct involvement, due to a higher incidence of invasive cancer (43.1% *versus* 17.7%) [16,17]. In the literature, only a few reports are found on patients who underwent pancreatic surgery after liver transplantation while the number of liver transplant recipients undergoing major pancreatic surgery for IPMN seems even scarcer. A recent retrospective study including 3,196 patients who underwent liver transplantation at the University of Pittsburgh Medical Center during a period of 7.5 years demonstrated that 0.6% required pancreatic surgery in the postoperative course of transplantation [18]. Of those, merely one patient underwent distal pancreatosplenectomy for IPMN while six patients underwent pancreatectomy for malignant tumors. Stauffer *et al*. described a series of 17 patients who were referred to pancreatic surgery prior to, simultaneously, or after liver transplantation and reported only one patient who underwent a pancreaticoduodenectomy for *de novo* pancreatic ductal adenocarcinoma 3.8 years after liver transplantation [19]. Other authors reported single cases of new onset pancreatic cancer in patients after liver transplantation [20,21]. Del Chiaro *et al*. presented a patient who was successfully treated for a BD-IPMN through enucleation of the cyst 5 years post-liver transplantation [22]. Lennon *et al*. recently conducted a monocentric study to determine the risk of progression in patient with IPMNs after liver transplantation. In this retrospective analysis, 23 liver transplant recipients with BD-IPMN were compared with a control group of 274 patients who did not undergo liver transplantation and with no history of immunosuppression [23]. Herein, 17.4% of the liver transplant patient and 16.4% in the control group developed high-risk features during the follow-up program. Therefore, no significant increase in the risk of progression of BD-IPMNs was found in patients after liver transplantation.

Prospective observation of liver transplant recipients focusing on cystic pancreatic changes in correlation with their clinical course will be essential to understand the conundrum of cyst formation in this patient cohort. Until we gather more information to develop evidence-based guidelines, we recommend careful individual management of every patient and to discuss any treatment in an experienced interdisciplinary tumor board along with Sendai recommendations.

## Conclusions

Information on an increased occurrence of cystic lesions such as IPMN after liver transplantation and a possible etiological coherence is still sparse. From limited data available and our experience, there is no increased risk for progression towards invasive adenocarcinoma in this population. Thus, we recommend a critical and individual patient-by-patient evaluation in the therapeutic triage while recommendations from the Sendai consensus criteria should be respected. Patients should be treated in a tertiary referral center for hepatopancreatobiliary and transplant surgery/medicine.

## Consent

Written informed consent was obtained from the patient for publication of this case report and any accompanying images. A copy of the written consent is available for review by the Editor-in-Chief of this journal.

## Abbreviations

BD-IPMN: Branch duct IPMN
CT: Computed tomography
ERCP: Endoscopic retrograde cholangiopancreatography
IPMN: Intraductal papillary mucinous neoplasm
MRCP: Magnetic resonance cholangiopancreatography
PTLD: Post-transplant lymphoproliferative disorder
SIR: Standardized incidence ratio
