# Erratum to: Extensive multifocal branch duct IPMN of the pancreas after liver transplantation: is surgery justified?

**DOI:** 10.1186/s40001-015-0143-3

**Published:** 2015-05-20

**Authors:** Vittorio Branchi, Philipp Lingohr, Winfried A. Willinek, Edwin Bölke, Alexander Semaan, Hui Zhou, Glen Kristiansen, Günter Klöppel, Jörg C Kalff, Nico Schäfer, Hanno Matthaei

**Affiliations:** Department of Surgery, University Hospital of Bonn, Bonn, Germany; Institute of Pathology, University Hospital of Bonn, Bonn, Germany; Department of Radiology, University Hospital of Bonn, Bonn, Germany; Center for Integrated Oncology (CIO) Cologne-Bonn, University of Dusseldorf, Dusseldorf, Germany; Department of Radiation Oncology, University of Dusseldorf, Dusseldorf, Germany; Center for Pancreatic and Endocrine Tumors, Department of Pathology, Technical University Munich, Munich, Germany

## Erratum

After publication of this work [1], we noted that we inadvertently failed to include the complete list of all co-authors. The full list of authors has now been updated. The Authors' contributions and Competing interests section modified accordingly. We are publishing this erratum to update the author list, which is as follows:

Vittorio Branchi, Philipp Lingohr, Winfried A Willinek, Edwin Bölke, Alexander Semaan, Hui Zhou, Glen Kristiansen, Günter Klöppel, Jörg C Kalff, Nico Schäfer and Hanno Matthaei
